# Canine candidate genes for dilated cardiomyopathy: annotation of and polymorphic markers for 14 genes

**DOI:** 10.1186/1746-6148-3-28

**Published:** 2007-10-19

**Authors:** Anje C Wiersma, Peter AJ Leegwater, Bernard A van Oost, William E Ollier, Joanna Dukes-McEwan

**Affiliations:** 1 Small Animal Teaching Hospital, University of Liverpool, Leahurst, Chester High Road, Neston, CH64 7TE, UK; 2 Centre for Integrated Genomic Medical Research, Division of Epidemiology and Health Sciences, The University of Manchester, Manchester, M13 9PT, UK; 3 Department of Clinical Sciences of Companion Animals, Faculty of Veterinary Medicine, Utrecht University, 3508 TD Utrecht, The Netherlands; 4 American University of the Caribbean, Department of Molecular Cell Biology, #1 University Drive at Jordan Road, Cupecoy, St.Maarten, Dutch Antilles

## Abstract

**Background:**

Dilated cardiomyopathy is a myocardial disease occurring in humans and domestic animals and is characterized by dilatation of the left ventricle, reduced systolic function and increased sphericity of the left ventricle. Dilated cardiomyopathy has been observed in several, mostly large and giant, dog breeds, such as the Dobermann and the Great Dane. A number of genes have been identified, which are associated with dilated cardiomyopathy in the human, mouse and hamster. These genes mainly encode structural proteins of the cardiac myocyte.

**Results:**

We present the annotation of, and marker development for, 14 of these genes of the dog genome, i.e. α-cardiac actin, caveolin 1, cysteine-rich protein 3, desmin, lamin A/C, LIM-domain binding factor 3, myosin heavy polypeptide 7, phospholamban, sarcoglycan δ, titin cap, α-tropomyosin, troponin I, troponin T and vinculin. A total of 33 Single Nucleotide Polymorphisms were identified for these canine genes and 11 polymorphic microsatellite repeats were developed.

**Conclusion:**

The presented polymorphisms provide a tool to investigate the role of the corresponding genes in canine Dilated Cardiomyopathy by linkage analysis or association studies.

## Background

Dilated cardiomyopathy (DCM) is a myocardial disease characterized by dilatation of the left ventricle, reduced systolic function and increased sphericity of the left ventricle. This disease has been described in different species and multiple genes have been found in the human [1], mouse [2] and hamster [3] causing DCM. These genes mainly encode cyto-skeletal components of the cardiac myocytes and can be divided into sarcomeric and extra-sarcomeric proteins. The identified sarcomeric proteins involved in DCM include α-cardiac actin, encoded by *ACTC *[4], cysteine-rich protein 3 (*CSRP3*) [5], LIM-domain binding factor 3 (*LDB3*, also known as *Cypher *or *ZASP*) [6], myosin heavy polypeptide 7 (*MYH7*) [7], titin cap (*TCAP*) [8], α-tropomyosin (*TPM1*), troponin I (*TNNI3*) [9], troponin T (*TNNT2*) [7], titin (*TTN*) [10] and vinculin (*VCL*) [11]. The extra-sarcomeric proteins implicated in DCM are encoded by the genes including caveolin 1 (*CAV1*) [2], desmin (*DES*) [12], lamin A/C (*LMNA*) [13], phospholamban (*PLN*) [14] and sarcoglycan δ (*SGCD*) [3]. The genes encoding all of the above proteins are located on the autosomal chromosomes. X-linked genes implicated in DCM include dystrophin (*DYS*) [15] and tafazzin (*TAZ*) [16]. In addition, mitochondrial dysfunction and mitochondrial DNA (mtDNA) mutations have been associated with maternally inherited DCM [17]. Furthermore, DCM has also been described with arrhythmias, with mutations in genes encoding sodium [18] and potassium channels [19].

DCM has been described in many different breeds of mostly giant and large dogs, including the Dobermann [20], Great Dane [21], Newfoundland [22] and Irish Wolfhound [23]. Clinical variation exists in the presentation and progression of DCM between different dog breeds and breed specific variation has also been found in histological findings in DCM-affected hearts tissue [24]. Since clinical DCM may be a late onset disease, following a long pre-symptomatic course, dogs are often used for breeding before the disease becomes apparent [25]. So far, no causative mutation has been discovered in canine DCM. The phenotype of the adult onset forms of canine DCM in most breeds is consistent with a defect in components of the cytoskeleton.

Of the 14 autosomal DCM candidate genes for the dog, *ACTC*, *CAV1*, *CSRP3*, *DES*, *LDB3*, *LMNA*, *MYH7*, *PLN*, *SGCD*, *TCAP*, *TNNI3*, *TNNT2*, *TPM1 *and *VCL*, genomic information and/or polymorphic markers were already available for *ACTC *[26,27], *DES *[28], *PLN *[29], *SGCD *[30] and *TPM1 *[31]. In this article, we describe a complete set of polymorphic markers for these 14 candidate genes for canine DCM. The markers, both microsatellites and Single Nucleotide Polymorphisms (SNPs), provide a useful tool to perform linkage and association studies between each of these genes and DCM in the different dog breeds. Furthermore, we present the annotation of 14 candidate genes in the canine genome, which will facilitate mutation screening of these genes.

## Genomic Annotation

The 14 canine DCM candidate genes were identified on the canine genome by means of a BLAST analysis [32], using available canine and human DNA sequences as a query (Table 1). The exons were identified based on the corresponding human exon sequence (retrieved from [33], Table 1). Each gene was found to be covered by 1 to 5 contigs of the *Canis familiaris *genome build 1.1. (Additional file 1 and Table 1). *CAV1 *was covered by 2 neighbouring contigs and the 3 coding exons matched the human ones. Exon 1 of the dog seemed to have an extra nucleotide (T, position 336 of [Genbank: AAEX01048547]) compared to human exon 1 of *CAV1*. However, this nucleotide was not present in the single trace file of the *Canis familiaris *Trace Archive [34] covering this sequence. Canine DES had 1 amino acid less than the human protein. The canine LDB3 protein is 67 amino acids shorter than human LDB3. Canine LMNA had 1 amino acid extra compared to the human protein. Exon 24 of canine *MYH7 *seemed to have 1 bp extra (G, bp 7,902 of [Genbank: AAEX01041100]), however, this nucleotide was not present in any of 11 *Canis familiaris *trace sequences covering this position. Without this extra nucleotide, canine exon 24 matched the human exon. Canine TNNI3 had 1 amino acid extra compared to the human protein. For *TNNT2*, coding exons 1, 15 and 16 could not be recognized in canine genomic contigs. *TNNT2 *exon 6 showed 1 extra bp compared to human (G, bp 5622 of [Genbank: AAEX01013360]), however, this nucleotide was not found in the 2 traces covering this DNA sequence. Without this additional bp, exon 6 matched the corresponding human exon exactly in length. Exon 12 had 1 codon less than the human gene. Exon 13 was located at the end of genomic contig [Genbank: AAEX01013360] and although its terminal 2 putative bp were not included in this contig, exon 12 seemed to match the human exon. For the remaining candidate genes, *ACTC*, *CSRP3*, *PLN*, *SGCD*, *TCAP*, *TPM1 *and *VCL*, the annotated canine exons matched the corresponding human exons exactly. We could not identify non-coding exons. Apparently, the conservation of these exons is too low for identification purposes. Complementary DNA sequencing is necessary to identify these non-coding exons. All of the predicted introns of the 14 candidate genes started and ended with the canonical GT and AG dinucleotides, respectively [35]. Even though a high quality DNA sequence of the canine genome has recently become available, it has not yet been fully annotated.

**Table 1:** Assignment, genomic location and the degree of sequence conservation compared to human of the canine DCM candidate genes.

**Gene**	**Annotation of canine gene**				**CFA**	**Similarity to human ^4 ^**
			
	**Identification sequence ^1^**	**ENST 00000..^2^**	**AAEX010... ^3^**	**Dog prot. (a.a.)**		
***ACTC***	AF203019 (C),	290378	13478	377	30	100% AAB59619
	AF203020 (C)					
***CAV1***	U47060 (C)	341049	48546, 48547	178	14	96% NP_001744
***CSRP3***	BC024010 (H)	265968	17412	194	21	99% AAH24010
***DES***	BK005142 (C)	273074	55032	469	37	97% NP_001918
***LDB3***	NM_007078 (H)	361816	16582, 16583^(5)^, 16584	660	4	79% AB014513
***LMNA***	AF427092 (C)	310777	12733, 12734	665	7	98% CAI15522
***MYH7***	NM_000257 (H)	355349	41099, 41100	1935	8	98% NP_000248
***PLN***	Y00399 (C)	357525	14037	52	1	96% CAI21610
***SGCD***	NM_000337 (H)	303006	16848, 16849^(5)^, 16850^(5)^, 16851, 16852	289	4	98% NP_000328
***TCAP***	NM_003673 (H)	309889	22011	167	9	90% CAA09479
***TNNI3***	AF506750 (C)	344887	53923	211	1	95% CAG46782
***TNNT2*** **(ex 2–14)**	NM_000364 (H)	367317	13359, 13360	254	7	90% NP_000355
***TPM1***	NM_000366 (H)	288398	08742	284	30	99% AAH07433
***VCL***	NM_003373 (H)	211998	16404	1134	4	99% NP_054706

^1 ^Sequence used to identify the canine gene in the dog genome, Genbank accession numbers; C = canine sequence, H = human sequence; ^2 ^Transcript ID numbers of human annotation [33] used to annotate the canine gene; ^3 ^canine genomic contig in which the gene's coding exons were identified; ^4 ^the percentage identity of each canine protein compared to the human protein (Genbank accession number is listed); ^5 ^canine genomic contig containing only intronic sequence.

The conservation of the coding region of each gene was assessed by BLAST comparison of the cDNA and derived amino acid sequences with those of human (at the website of NCBI [36], BLASTN and TBLASTX analysis, respectively). The percentages of identity at the nucleotide level varied between 88 and 95% (Table 1). At the amino acid level, the percentages of identity varied in general between 90–100%, except for the canine LDB3 protein, that was 79% identical to the human protein. The canine ACTC protein appeared to be identical to the human protein. In *LDB3*, a relatively low percentage of identity was found between the canine and human gene, both at the cDNA and the protein level. This was caused by the large (inframe) loss of part of exons (i.e. 4, 7, 8 and 9) compared to the human gene: the canine gene had 660 codons, the human gene had 734 codons.

The chromosomal position of the 14 canine candidate genes can be found in Table 1.

When analysing the location of the genes in the dog genome (Table 1), using the canine-human comparative map of Guyon et al. [37], each was found to be syntenic to the human location.

## Polymorphisms

### Single Nucleotide Polymorphism detection

We used denaturing high-performance liquid chromatography (DHPLC) analysis for the detection of SNPs in amplified genomic canine DNA fragments. Polymorphisms were assessed in DNA from Newfoundland dogs. For each gene, several DNA fragments of approximately 500 bp were selected based on melting profile (analyzed with WAVEMAKER™ software from Transgenomic) with a maximum of 2 melting temperatures covering each product. The melting behaviour of a fragment depends on the fragment's DNA sequence. Primers were designed using Primer3 [38] and annealing temperatures of the PCRs were optimized (Table 2). Touchdown PCR amplification of these fragments was performed with DNA of Newfoundland dogs (n = 16; 8 unrelated founders of a pedigree of Newfoundland dogs and 8 family members), using HotStartTaq DNA Polymerase (Qiagen). The Touchdown (TD) PCR program consisted of a denaturing step of 5 min at 95°C, followed by 14 cycles of 95°C 30 sec, Ta +7°C 30 sec, 72°C 20 sec, with a Ta decrease of 0.5°C/cycle, followed by 25 cycles of 30 sec at 94°C, 30 sec at Ta°C, 30 sec at 72°C, followed by a final extension at 72°C for 2 min (Ta in Table 2). Subsequently, a heteroduplex formation step was carried out to allow formation of hetero- and homo-duplex products; the PCR products were heated 5 min at 95°C, after which the temperature was decreased gradually (38 cycles of 1 min, temperature decreasing 1.5°C/cycle), followed by a final step of 5 min at 10°C. Mutation analysis of the PCR products, based on the presence of heteroduplexes, followed on a WAVE instrument (WAVE Nucleic Acid Fragment Analysis System, Transgenomic). Multiple WAVE patterns of a single PCR fragment in different dogs pointed at existence of both homoduplexes and heteroduplexes and, therefore, indicated potential presence of SNPs in the fragment. In that case, the PCR fragment (of at least of 2 dogs per WAVE pattern) was cleaned (Shrimp Alkaline Phosphatase/ExoI) and the DNA sequence was obtained to determine the identity of the SNPs, by a commercial company (Lark Technologies™, UK).

**Table 2:** Single Nucleotide Polymorphisms in the DCM candidate genes. For each SNP its origin, its primers and the PCR conditions, and its informativity are listed.

**Gene**	**dbSNP** ** access. no. ** **ss4985...**	**SNP**	**Primers (5'-3')** ** Forward;** ** Reverse**	**Ta (°C)^1^**	**Prod. size (bp)**	**Informativeness^2^**	
						
						**PIC**	**#chr**
***ACTC***	2973	5,452 G/A^a,3,4^	gccctggattttgagaatgagat acgatcagcaataccagggtaca	62.0 ^1^	1067	0.14	12
***CAV1***	2978	30,312 A/G ^b,5^	tgagtgccttgcttgtgg gcatcattggaacttgttgg	62.0	565	0.28	24
	2979	30,088 G/A^5^	tgagtgccttgcttgtgg gcatcattggaacttgttgg	62.0	565	0.24	24
***CSPR3***	2980	31,216 A/G ^6^	ggaggccaggatgagaac gtttattgtactgaatgatggtcag	62.0	507	0.15	22
	2981	25,753 T/C ^6^	aatcatcctcccattgttcc cagaagtgctcatagtctttaccc	58.0	510	0.37	24
	2982	25,446 A/G ^6^	aatcatcctcccattgttcc cagaagtgctcatagtctttaccc	58.0	510	0.24	24
	2983	28,779 A/G ^6^	atggacctttgtatctccag tctgtaggtttcattcattgg	58.0	455	0.19	24
	2984	28,742 C/A ^6^	atggacctttgtatctccag tctgtaggtttcattcattgg	58.0	455	0.19	24
	2985	28,737 G/A ^6^	atggacctttgtatctccag tctgtaggtttcattcattgg	58.0	455	0.19	24
	2986	28,642 T/A ^6^	atggacctttgtatctccag tctgtaggtttcattcattgg	58.0	455	0.19	24
***DES***	2989	15,228 C/T ^7^	cgtcacaacccccacaag gctgggtgccatgaggtc	67.0	530	0.30	8
	2990	15,224 C/G ^7^	cgtcacaacccccacaag gctgggtgccatgaggtc	67.0	530	0.19	8
	2991	15,166 G/A ^7^	cgtcacaacccccacaag gctgggtgccatgaggtc	67.0	530	0.19	8
	2992	15,006 C/T ^7^	cgtcacaacccccacaag gctgggtgccatgaggtc	67.0	530	0.19	8
	2993	19,903 T/C ^7^	agggcagagggagaccag gacctaatggtgggctttacc	66.0	575	0.30	8
	2975	19,196 C/T ^c,7,8^	ttgcttgaccactaccagga^9^ agatgttcttagccgcgatg^10^	57.0 ^1^	402	0.35	12
	2976	19,105 G/A ^7,8^	ttgcttgaccactaccagga^9^ agatgttcttagccgcgatg^10^	57.0 ^1^	402	0.30	12
***LDB3***	2987	14,090 C/T ^11^	tgttaatcacctctgcggatagt ggctccctacacgttgatg	58.0	540	0.33	24
	2988	25,205 T/C ^12,d^	gcctcctccatcctgacc cctcccagtaccctgtaggc	66.0	566	0.19	24
	s2974	25,452 A/G ^12^	gcctcctccatcctgacc cctcccagtaccctgtaggc	66.0	566	0.38	24
***PLN***	2994	51,818 A/G ^13^	tggtttgccttcatacactacaac tgtcttcatctgtgggattttg	64.0	573	0.21	14
***SGCD***	2995	30,703 G/C ^14^	ccttcagacccccatctagg ccacctgacataatcccactttag	66.0	521	0.36	8
	2996	151,312 A/G ^14^	ggaggtagcaaagtatagtgctc atgttcatgccaacaagc	62.0	558	0.30	8
	2997	29,656 C/G ^14^	ttccagccaactgagaagc cactgtcatttccatgtcaacc	58.0	525	0.30	8
	2998	116,470 A/G ^14^	gcaatctcctcctccagacc tcatggcctcactctgatctc	58.0	529	0.38	8
***TCAP***	2999	28,606 C/T ^15,e^	gctgcttcccttgaatgc cagacagtggcaggaatcg	64.0	588	0.28	24
	2977	29,957 T/C ^15,f^	gtagagggtagcagatttcagg ctctgggcaaactacaaagc	69.0	555	0.26	16
	3000	30,330 A/G ^15,g^	tgctttgtagtttgcccagag agccagccaccctgtttac	64.0	557	0.30	8
	3001	30,687 C/T ^15,h^	tgctttgtagtttgcccagag agccagccaccctgtttac	64.0	557	0.30	8
***TNNT2***	3002	10,466 C/T ^16^	tgaccctcacttggggaac cgcagggctcttccagac	58.0	519	0.38	24
	3003	10,577 T/C ^16^	tgaccctcacttggggaac cgcagggctcttccagac	58.0	519	0.38	24
	3004	10,671 T/C ^16^	tgaccctcacttggggaac cgcagggctcttccagac	58.0	519	0.38	24
***VCL***	3005	177,743 G/A^17^	tgcaggccacagagatgc ggaatgagggcggagcag	62.0	491	0.30	8

^1 ^All PCR program were Touchdown (TD) at the listed Ta, accept for the *ACTC *SNP: 94°C 5 min, 35× (94°C 30 sec, 62°C 1 min, 72°C 1 min), 72°C 10 min, 20°C ∞; and for the *DES *SNPs 19,196 C/T and 19,105 G/A: 94°C 10 min, 35× (94°C 30 sec, 57°C 30 sec, 72°30 sec), 72°C 10 min, 20°∞; ^2 ^The informativeness of each SNP was described by its polymorphism information content (PIC), based on the number of genotyped chromosomes (#chr) listed; ^3 ^SNP detected while sequencing available *ACTC *SNPs (166C/T and 38C/T of [Genbank: AF203019] and 289T/A of [Genbank: AF203020], these were in the dog genome, respectively, 4,871G/A, 5,000 G/A and 5,454 A/T in [Genbank: AAEX01013478)] [26]; ^4 ^in genomic contig AAEX01013478; ^5 ^AAEX01048546; ^6 ^AAEX01017412; ^7 ^AAEX01055032; ^8 ^SNP detected while sequencing available *DES *SNPs (1,808C/T and 1,851G/C of [Genbank: BK005142], these were in the dog genome, respectively, 19,262 G/A and 19,218C/G in [Genbank: AAEX01055032]) [28]; ^9 ^M13-tailed F-primer: 5'- GTTTTCCCAGTCACGAC---- 3'; ^10 ^M13-tailed R-primer: 5'- CAGGAAACAGCTATGAC----3'; ^11 ^in genomic contig AAEX01016584; ^12 ^AAEX01016582; ^13 ^AAEX01014037; ^14 ^AAEX01016848; ^15 ^AAEX01022011; ^16 ^AAEX01013360; ^17 ^AAEX01016404; ^a ^is identical to SNP BICF237J37997 (Broad, at [39]); ^b ^identical to BICFPJ1220038; ^c ^identical to BICFPJ152241; ^d ^identical to BICF231J18538; ^e ^identical to BICFG630J165218; ^f ^identical to BIFG630J165217; ^g ^identical to BICFG630J165215; ^h ^identical to BICFG630J165213.

Twenty-eight SNPs were discovered by WAVE analysis (Table 2). No indication of the presence of a SNP was found in WAVE fragments of *LMNA*, *MYH7 *and *TNNI3 *(3, 5 and 3 fragments analyzed, respectively). One new SNP, TCAP SNP 29,957 T/C in genomic contig [Genbank: AAEX01022011], was found when we resequenced a *TCAP *fragment in a group of Newfoundland dogs. WAVE analysis of this fragment had not indicated presence of a potential SNP – although the obtained DNA sequences showed that both homozygous and heterozygous animals were among the dogs used for WAVE analysis. Conversely, sometimes WAVE analysis indicated potential presence of SNPs, yet sequencing of dogs with different WAVE patterns did not confirm these. This could be due to the sequencing procedure used.

In search of additional SNPs for canine *ACTC *and *DES*, genomic DNA fragments containing SNPs annotated by others (Table 2) were resequenced. After PCR amplification of these fragments, 1 μl of 1:15 diluted PCR product was used in a Tercycle big dye reaction with the F-PCR-primer for the ACTC SNP and a HPLC-purified M13 F-primer (5'-GTTTTCCCAGTCACGAC-3') for the DES SNPs. The Tercycle consisted of 25 cycles of 30 sec at 96°C, 15 sec at 55°C and 2 min at 60°C. After purification (Sephadex TM G50 Superfine, Amersham Biosciences), each product was processed with an ABI PRISM^® ^3100 Genetic Analyzer (Applied Biosystems). Five SNPs (*ACTC *5,452G/A; *DES *19,196C/T and 19,105G/A; *LDB3 *25,452A/G and *TCAP *29.957 T/C) were identified by resequencing areas of earlier described SNPs (Table 2).

Of the total of 33 identified SNPs, 4 were in coding regions (*DES *15,006C/T, *LDB3 *14,090C/T, *TCAP *29,957T/C and *TNNT2 *10,466C/T). These exonic SNPs, however, did not cause polymorphisms at the amino acid level. Comparing the 33 newly discovered SNPs to the dog SNP database of the Broad Institute [39] showed 25 of our SNPs to be new, the remaining 8 SNPs matched SNPs present in the Broad database (see Table 2). This indicates that, in addition to the many SNPs that have become available by random sequencing of the dog genome, many more canine SNPs exist. Our limited search for SNPs in 14 DCM candidate genes took place in a single breed, the Newfoundland dog. However, a high percentage of SNPs found in one breed can be expected to be polymorphic in other breeds too [40]. All identified SNPs were submitted to dbSNP and the respective accession numbers are listed in Table 2.

### Detection of microsatellite polymorphisms

Simple DNA sequences composed of CA, GAAA or GA repeats were identified in the genomic contigs that contain the candidate genes or in neighbouring contigs. For *VCL*, a polymorphic microsatellite became available through personal communication with P.Stabej (Table 3; a repeat was obtained from BAC RP81-251B5, isolated using methods as described in [28] with an overgo probe based on murine *VCL *exon 17, F-overgo CCAAGGTCAGAGAAGCCTTCCAAC, R-overgo AAGTCAGGCTCCTGAGGTTGGAAG). Primers were designed from the DNA sequence flanking the repeats and the forward primer was fluorescently labelled with 6-FAM or HEX. For some microsatellites, a 3-primer protocol was used for the PCR amplification (Table 3), using an M13-tailed (GTTTTCCCAGTCACGAC----- (5'-3')) F-primer, a 6-FAM-labelled M13 primer (GTTTTCCCAGTCACGAC (5'-3')) and a R-primer. Genotyping PCR reactions were incubated 12 min at 94°C, followed by 35 cycles of 10 sec at 94°C, 15 sec at Ta°C and 30 sec at 72°C, and a final step of 20 min at 72°C (Ta in Table 3). An ABI PRISM ^® ^3100 Genetic Analyzer (Applied Biosystems) was used for genotyping and allele sizes were determined with Genescan Analysis 3.7 and Genotyper 3.7 software (Applied Biosystems). Eleven polymorphic microsatellites were developed for *ACTC *(2 markers), *CAV1 *(1), *CSRP3 *(1), *LMNA *(2), *MYH7 *(1), *TNNI3 *(3) and *VCL *(1) (Table 3). The markers, mostly CA-repeats, showed multiple allele sizes (2–6 alleles/marker) in a group of 16 Newfoundland dogs (Table 3). To describe the informativeness of our microsatellite markers, the polymorphism information content (PIC) was obtained based on the genotypes of unrelated founders of a family of Newfoundland dogs (Table 3). According to [41], 2 of the 11 newly designed microsatellites were considered highly informative (PIC>0.50), 7 reasonable informative (0.25<PIC<0.50) and 2 slightly informative (PIC<0.25) in the Newfoundland founder dogs. Besides the 11 polymorphic microsatellites, 2 other markers were found to be monomorphic in the group of Newfoundland dogs, but might be polymorphic in other breeds. This was a *MYH7 *CA-repeat (position 11,730 of [Genbank: AAEX010141100]) and a *TNNI3 *CA-repeat (position 17,739 of [Genbank: AAEX01053915]. An already available microsatellite for TPM1 [31] was shown to be highly informative in our group (Table 3).

**Table 3:** Polymorphic microsatellite markers for canine DCM candidate genes^1^

**Gene**	**Repeat**	**Primers (5'-3')** **Forward;** ** Reverse**	**Ta (°C**)	**Detected alleles**		**Informativeness^2^**		**Origin (bp ... of contig AAEX 010...)**	**Distance to gene^3^**
							
				**bp**	#	**PIC**	**#chr**		
*ACTC*	15CA	actccgaagaaggaagtcaac^4^ gttcccatctatgagggctat	57.0	234–238	2	0.17	10	bp 37,720/..13479	69.2 kb downstr. Stop
	20CA	ggaacaaggtgctgttagacc^5^ cacattccaccgagtaggc^7^	59.0	338–356	5	0.42	24	bp 5,945/..13478	intragenic (intron 4)
*CAV1*	13CA	ccacagagctagaaagctacg^4^ tgttgcaaacaccctatgat	54.5	240–242	2	0.28	24	bp 39,315/...48546	8.2 kb downstr. Stop
*CSRP3*	15CA	catgtcctgcaagttaatggt^4^ ggatttctattctgggtttcc	53.0	237–245	3	0.41	24	bp 38,367/..17412	2.8 kb upstr. Start
*LMNA*	16CA	gggtggtagatgagcatttc^6^ gaagagaacaagtgggcaag	54.5	204–212	4	0.36	12	bp 5,128/..12734	9.3 kb downstr. Stop
	18GAAA	ggaagatgagactgttagaatgc^5^ caggccatgattacttttcc^7^	57.0	321–344	6	0.67	24	bp 26,754/..12735	22.6 kb downstr. Stop
*MYH7*	21CA	gatatcctgggattaaagactgg^5^ ctattttgccctcttcatgg^7^	58.0	351–363	4	0.37	24	bp 1,418/..41098	36.6 kb downstr. Stop
*TNNI3*	20CAa	tcaaacagggaaacctgaac^6^ gattattcagctcccagaacc^7^	57.0	297–301	3	0.38	24	bp 597/..53929	119.3 kb upstr. Start
	20CAb	ttccagttgattgtttctctgc^5 ^gcggtttagcactgcattc^7^	59.0	302–306	2	0.08	24	bp 13,248/..53916	110.2 kb downstr. Stop
	17GA	tccaacctcagggtactgg^5 ^catgccatggagctatgc^7^	59.0	304–312	3	0.37	24	bp 48,910/..53930	179.8 kb upstr. Start
*TPM1*	19CA^8^	actgtgtccagagtgcagcta^4^ gattgctagactggc	60.0	467–483	4	0.67	12	bp 88,113/..08742	6.5 kb downstr. Stop
*VCL*	15GAAA^9^	caatttcttttccaatcacattag ^10^ gccattttgcattctcttcaa	54.0	150–170	6	0.69	24	bp 12,680/..16406	88.6 kb downstr. Stop

^1 ^Microsatellites for *DES *and *SGCD *were demonstrated in, respectively, [28] and [30]; ^2 ^The informativeness of each SNP was described by its polymorphism information content (PIC), based on the number of genotyped chromosomes (#chr) listed; ^3 ^Based on genomic build 1.1; ^4 ^F-primer fluorescently labelled with 6FAM; ^5 ^Three-primer protocol used; ^6 ^F-primer fluorescently labelled with HEX; ^7 ^extra tail on R-primer: GTGTCTT---- (5'-3') to promote addition of an Adenosine residue at the 3'-end of the complementary DNA strand; ^8 ^Microsatellite demonstrated in [31]; ^9 ^Personal communication P.Stabej; ^10 ^F-primer fluorescently labelled with TET.

The distance between the microsatellite and the corresponding gene was derived from the dog genome build 1.1 [42] and can be found in Table 3. This distance varied from zero for an intragenic microsatellite to 179.8 kb. The genomic locations of polymorphic microsatellites, already available for *DES*, *SGCD*, *TPM1 *and *VCL*, were determined. For *DES *a CA-repeat [28] was located at position 5,688 of [Genbank: AAEX01055032], 9.0 kb downstream of the stop codon. For *SGCD *both a GAAA-repeat and a CA-repeat were available [30]. The first was located at position 76,364 of [Genbank: AAEX4801016848], the second at position 42,047 of the same genomic contig and both markers are in intron 7 of SGCD. For *TPM1 *a GA-repeat [31] was located at position 88,113 of [Genbank: AAEX01008742], 6.5 kb downstream of the stop codon. A polymorphic GAAA-repeat for *VCL *showed to be located at position 12,680 of [Genbank: AAEX01016406] in the dog genome, 88.6 kb downstream of the stop codon.

## Conclusion

With the annotation of these 14 candidate genes for DCM and the identification of polymorphic markers, the genes can be evaluated for the involvement in breed specific DCM. The SNPs and microsatellites presented in this paper are a powerful tool to analyse linkage between the fourteen candidate genes encoding cytoskeletal proteins and DCM in the dog. The annotation of each gene facilitates screening of these genes for mutations in naturally occurring canine DCM in specific breeds, potential models for forms of human DCM.

## Authors' contributions

ACW carried out the molecular genetic studies and drafted the manuscript. PAJL participated in the design of the study, helped to draft the manuscript. BAvO participated in the design of the study and was co-applicant for funding. WEO participated in the coordination of the study. JDMcE conceived of the study and was main applicant for funding. She phenotyped the dogs, collected the samples and extracted the genomic DNA.

All authors had read and approved the final manuscript.

## Supplementary Material

Additional file 1 Overview of the genomic organization of the canine *ACTC *(A), *CAV1 *(B), *CSRP3 *(C), *DES *(D), *LDB3 *(E), *LMNA *(F), *MYH7 *(G), *PLN *(H), *SGCD *(J), *TCAP *(K), *TNN-I3 *(L), *TNN-T2 *(M), *TPM1 *(N) and *VCL *(P) gene, in build 1.1 of the canine genome. The size of each coding exon, its actual location in bp in the respective genomic contig, 10 bp of DNA sequence at the 5'end and 3'end of the exon, 10 bp of the flanking intron and the intron sizes are listed. In case the coding sequence of a gene was covered by multiple *Canis familiaris *genomic contigs, the size of the intron covered by more than one contig was based on information of the respective chromosome. For the exons containing the start and the stop codon, the number of coding bp is listed as ORF (open reading frame); the location of the respective codon is listed between brackets. Click here for file
