# Supplementary material for: Canine candidate genes for dilated cardiomyopathy: annotation of and polymorphic markers for 14 genes
Source: BMC Vet Res. 2007 Oct 19;3:28. doi: 10.1186/1746-6148-3-28 (PMC2194671; doi:10.1186/1746-6148-3-28)
Supplement: Additional file 1 — Overview of the genomic organization of the canine ACTC (A), CAV1 (B), CSRP3 (C), DES (D), LDB3 (E), LMNA (F), MYH7 (G), PLN (H), SGCD (J), TCAP (K), TNN-I3 (L), TNN-T2 (M), TPM1 (N) and VCL (P) gene, in build 1.1 of the canine genome. The size of each coding exon, its actual location in bp in the respective genomic contig, 10 bp of DNA sequence at the 5'end and 3'end of the exon, 10 bp of the flanking intron and the intron sizes are listed. In case the coding sequence of a gene was covered by multiple Canis familiaris genomic contigs, the size of the intron covered by more than one contig was based on information of the respective chromosome. For the exons containing the start and the stop codon, the number of coding bp is listed as ORF (open reading frame); the location of the respective codon is listed between brackets. [file 1746-6148-3-28-S1.doc]

# Additional file 1

Overview of the genomic organization of the canine *ACTC* (A), *CAV1* (B), *CSRP3* (C), *DES* (D), *LDB3* (E), *LMNA* (F), *MYH7* (G), *PLN* (H), *SGCD* (J), *TCAP* (K), *TNN-I3* (L), *TNN-T2* (M), *TPM1* (N) and *VCL* (P) gene, in build 1.1 of the canine genome

The size of each coding exon, its actual location in bp in the respective genomic contig, 10 bp of DNA sequence at the 5’end and 3’end of the exon, 10 bp of the flanking intron and the intron sizes are listed. In case the coding sequence of a gene was covered by multiple *Canis familiaris* genomic contigs, the size of the intron covered by more than one contig was based on information of the respective chromosome. For the exons containing the start and the stop codon, the number of coding bp is listed as ORF (open reading frame); the location of the respective codon is listed between brackets.

Additional file 1A

Intron-exon structure of canine *ACTC*

| Exon | Exon size | Location | | Gen.contig | Intron | 5'end exon | 3'end exon | Intron | Intron |
| --- | --- | --- | --- | --- | --- | --- | --- | --- | --- |
| no. | (bp) | from bp | to bp | AAEX010..... |  |  |  |  | size(Kb) |
| 2 | 129 ORF | (8,356) | 8,228 | 13478 |  |  | …gcgccaccag | gtagacgccg… | 1.116 |
| 3 | 325 | 7,111 | 6,787 | 13478 | …cttcttacag | ggagtcatgg… | …cgtaccaccg | gtatgtttgg… | 0.638 |
| 4 | 162 | 6,148 | 5,987 | 13478 | …ttcctgacag | gcattgttct… | …gtcaccactg | gtgagtgtgt… | 0.144 |
| 5 | 192 | 5,842 | 5,651 | 13478 | …atttctatag | ctgaacgtga… | …tcctttatcg | gtgagtggta… | 0.790 |
| 6 | 182 | 4,860 | 4,679 | 13478 | …ttatttctag | gtatggaatc… | …gaagattaag | gtaaagaact… | 0.499 |
| 7 | 144 ORF | 4,179 | (4,035) | 13478 | …ctttctgcag | atcattgctc… |  |  |  |

Additional file 1B

Intron-exon structure of canine *CAV1*

| Exon | Exon size | Location | | Gen.contig | Intron | 5'end exon | 3'end exon | Intron | Intron |
| --- | --- | --- | --- | --- | --- | --- | --- | --- | --- |
| no. | (bp) | from bp | to bp | AAEX010..... |  |  |  |  | size(Kb) |
| 1 | 30 ORF^1^ | (329) | 359 | 48547 |  |  | …agactccgag | gtaggctcgc… | 1.366^2^ |
| 2 | 165 | 756 | 920 | 48546 | …ccccgcgcag | gggcacctct… | …cgtggtcaag | gtaggggagg… | 29.813 |
| 3 | 342 ORF | 30,734 | (31,075) | 48546 | …ttccttgcag | attgattttg… |  |  |  |

^1^according to build 1.1 of the canine genome, the ORF of exon 1 counted 31 bp. However, bp T at position 336 of [Genbank: AAEX01048547] (exon 1) was not present in the single *Canis familiaris* trace sequence covering this position, it disturbed the reading frame and did not seem to be correct. Therefore, the assumed size of exon 1 was 30 bp ORF; ^2^the estimated distance between [Genbank: AAEX01048547] and [Genbank: AAEX01048546] was 1 bp.

Additional file 1C

Intron-exon structure of canine *CSRP3*

| Exon | Exon size | Location | | Gen.contig | Intron | 5'end exon | 3'end exon | Intron | Intron |
| --- | --- | --- | --- | --- | --- | --- | --- | --- | --- |
| no. | (bp) | from bp | to bp | AAEX010..... |  |  |  |  | size(Kb) |
| 2 | 112 ORF | (35,605) | 35,494 | 17412 |  |  | …ttccactgca | gtgagttggg… | 4.756 |
| 3 | 169 | 30,737 | 30,569 | 17412 | …tccttggcag | tggcctgcag… | …agttccaaca | gtgagtcact… | 1.646 |
| 4 | 133 | 28,923 | 28,791 | 17412 | …ttcccaccag | gtccccaaag… | …aggcggtaag | gtaaggactg… | 1.149 |
| 5 | 94 | 27,641 | 27,548 | 17412 | …cgccccccag | ccttggcaca… | …tactgcaaag | gtgagtggtt… | 1.503 |
| 6 | 77 ORF | 26,044 | (25,968) | 17412 | …tcgttcacag | tttgctacgc… |  |  |  |

Additional file 1D

Intron-exon structure of canine *DES*

| Exon | Exon size | Location | | Gen.contig | Intron | 5'end exon | 3'end exon | Intron | Intron |
| --- | --- | --- | --- | --- | --- | --- | --- | --- | --- |
| no. | (bp) | from bp | to bp | AAEX010..... |  |  |  |  | size(Kb) |
| 1 | 575 ORF | (21,067) | 20,493 | 55032 |  |  | …tcaaggccaa | gtgaggaccc… | 0.966 |
| 2 | 61 | 19,526 | 19,466 | 55032 | …ttgtggccag | gctgcaagag… | …cttccgagcg | gtgagccctc… | 0.114 |
| 3 | 96 | 19,351 | 19,256 | 55032 | …ccactaccag | gacgtggatg… | …gcatgaagag | gtatgccctc… | 0.155 |
| 4 | 162 | 19,100 | 18,939 | 55032 | …gcccccgcag | gagatccgag… | …caagtcgaag | gtgggcaacc… | 0.155 |
| 5 | 126 | 18,783 | 18,658 | 55032 | …ctgcccttag | gtgtccgacc… | …caagggcacc | gtgagtcccc… | 0.400 |
| 6 | 221 | 18,257 | 18,037 | 55032 | …tctcctgcag | aatgattccc… | …aggaaagccg | gtgaggggct… | 1.293 |
| 7 | 44 | 16,743 | 16,700 | 55032 | …cccctttcag | gatcaacctc… | …aacttccgag | gtgagtgcat… | 1.683 |
| 8 | 83 | 15,016 | 14,934 | 55032 | …tccgttacag | aaacaagccc… | …ggatggggag | gtgagccacc… | 0.206 |
| 9 | 42 ORF | 14,727 | (14,686) | 55032 | …ttctccccag | gttgtcagtg… |  |  |  |

Additional file 1E

Intron-exon structure of canine *LDB3*

| Exon | Exon size | Location | | Gen.contig | Intron | 5'end exon | 3'end exon | Intron | Intron |
| --- | --- | --- | --- | --- | --- | --- | --- | --- | --- |
| no. | (bp) | from bp | to bp | AAEX010..... |  |  |  |  | size(Kb) |
| 1 | 93 ORF | (16,483) | 16,575 | 16582 |  |  | …tatctcccgg | gtaagtgtgc… | 8.085 |
| 2 | 152 | 24,661 | 24,812 | 16582 | …atcctctcag | atcacaccag… | …cgctgcagaa | gtgagtggag… | 0.520 |
| 3 | 76 | 25,333 | 25,408 | 16582 | …gtttctccag | gtcgaagcgc… | …ccaccagaag | gtaggtgctg… | 1.352 |
| 4 | 275 | 26,761 | 27,035 | 16582 | …ccccggccag | gaccctgctg… | …tcccaggagg | gtaggtagcg… | 9.320 |
| 5 | 170 | 36,356 | 36,525 | 16582 | …cgccccgcag | gagcctccct… | …acagagtaca | gtaagtgagg… | 0.261 |
| 6 | 37 | 36,787 | 36,823 | 16582 | …ctacccacag | tgcaagaccc… | …ggaggtcaag | gtaagtgcct… | 13.221 |
| 7 | 174 | 47,113 | 47,286 | 16582 | …gcctcatcag | cacccccact… | …acagcccgag | gtaactgccc… | 2.758 |
| 8 | 143 | 50,045 | 50,187 | 16582 | …ttggtttcag | gccccaggcc… | …taccagccag | gtgagaggca… | 6.177^1^ |
| 9 | 355 | 899 | 1,253 | 16584 | …ggcttggcag | tgcccgcggc… | …acgtcatcag | gtacggtcag… | 0.954 |
| 10 | 181 | 2,208 | 2,388 | 16584 | …gctcccccag | gggccccttt… | …aatcatgggg | gtaagtgggc… | 0.532 |
| 11 | 121 | 2,921 | 3,041 | 16584 | …tttcccccag | gaagtgatgc… | …tgtgagaaag | gtaggaccac… | 5.262 |
| 12 | 116 | 8,304 | 8,419 | 16584 | …ttcatttcag | actacgtcaa… | …catctgcgca | gtatgtgttt… | 5.596 |
| 13 | 90 ORF | 14,016 | (14,105) | 16584 | …tctctcgcag | gtgtgccacg… |  |  |  |

^1^the estimated distance between [Genbank: AAEX01016582] and [Genbank: AAEX01016583] was 1,084 bp, the estimated distance between [Genbank: AAEX01016583] and [Genbank: AAEX01016584] was 50 bp.

Additional file 1F

Intron-exon structure of canine *LMNA*

| Exon | Exon size | Location | | Gen.contig | Intron | 5'end exon | 3'end exon | Intron | Intron |
| --- | --- | --- | --- | --- | --- | --- | --- | --- | --- |
| no. | (bp) | from bp | to bp | AAEX010..... |  |  |  |  | size(Kb) |
| 1 | 356 ORF | (5,771) | 5,416 | 12733 |  |  | …tcaaagcgcg | gtgagtgcgc… | 10.171^1^ |
| 2 | 157 | 20,943 | 20,787 | 12734 | …gcttccttag | caataccaag… | …ggtgaccaag | gtgaggcctc… | 2.088 |
| 3 | 126 | 18,698 | 18,573 | 12734 | …ttcctctcag | ctcgaggcag… | …ctacagtgag | gtagggactg… | 0.228 |
| 4 | 171 | 18,344 | 18,174 | 12734 | …cccaccccag | gagctgcgtg… | …ctctgccaag | gtgcttggtg… | 0.112 |
| 5 | 126 | 18,061 | 17,936 | 12734 | …tcccccaaag | ctggataacg… | …gcaaaagcag | gtgtgcccct… | 0.511 |
| 6 | 221 | 17,424 | 17,204 | 12734 | …cctcacccag | ctggcagcca… | …aggaggaaag | gtgggtcggg… | 0.104 |
| 7 | 226 | 17,099 | 16,874 | 12734 | …cccttgccag | gctgcgacta… | …gtccagtgag | gtaggcgccc… | 0.377 |
| 8 | 108 | 16,496 | 16,389 | 12734 | …ttgctggcag | gaccagtcca… | …ggtggtgacg | gtgagtggca… | 0.114 |
| 9 | 120 | 16,274 | 16,155 | 12734 | …gcctccctag | atctgggctg… | …cactggggaa | gtgagtatgc… | 0.388 |
| 10 | 90 | 15,766 | 15,677 | 12734 | …tccctggcag | gaggtggcca… | …tcaccaccat | gtgagtggca… | 0.696 |
| 11 | 270 | 14,980 | 14,711 | 12734 | …tccttctcag | ggctcccact… | …ccgaacccag | gtgagttgtc… | 0.282 |
| 12 | 27 ORF | 14,428 | (14,402) | 12734 | …tctctctcag | agcccccaga… |  |  |  |

^1^the estimated distance between [Genbank: AAEX01012734] and [Genbank: AAEX01012733] was 76 bp.

Additional file 1G

Intron-exon structure of canine *MYH7*

| Exon | Exon size | Location | | Gen.contig | Intron | 5'end exon | 3'end exon | Intron | Intron |
| --- | --- | --- | --- | --- | --- | --- | --- | --- | --- |
| no. | (bp) | from bp | to bp | AAEX010..... |  |  |  |  | size(Kb) |
| 3 | 201 ORF | (26,787) | 26,987 | 41099 |  |  | …gaatggcaag | gtgggtgaca… | 0.296 |
| 4 | 144 | 27,284 | 27,427 | 41099 | …gctgttgcag | acggtgaccg… | …gatgatctac | gtgagtgcgc… | 0.291 |
| 5 | 157 | 27,719 | 27,875 | 41099 | …tctacactag | acctactcgg… | …atgctgacag | gtgacaggcc… | 0.180 |
| 6 | 28 | 28,056 | 28,083 | 41099 | …ctcccaacag | acagagaaaa… | …tcctgatcac | gtgagtgtag… | 0.812^1^ |
| 7 | 109 | 177 | 285 | 41100 | …gtcccaacag | tggagaatct… | …ccccggaaag | gtaggcctgt… | 0.083 |
| 8 | 93 | 369 | 461 | 41100 | …acctctgcag | ggcactctgg… | …ctcccgcttt | gtaagtggtc… | 0.103 |
| 9 | 64 | 565 | 628 | 41100 | …ttctcgccag | gggaaattca… | …atagagacct | gtgagtgcca… | 0.336 |
| 10 | 99 | 965 | 1,063 | 41100 | …tctcttacag | accttctgga… | …gagctgctgg | gtgagtcaga… | 0.234 |
| 11 | 104 | 1,298 | 1,401 | 41100 | …ctcctcccag | acatgctgct… | …ggccactgat | gtaagtgtgt… | 0.651 |
| 12 | 139 | 2,053 | 2,191 | 41100 | …ctgcctacag | aatgcctttg… | …ggcactgaag | gtgggagtct… | 0.092 |
| 13 | 119 | 2,284 | 2,402 | 41100 | …ctctttccag | aggctgacaa… | …tgtccaacag | gtgggtcatg… | 0.104 |
| 14 | 150 | 2,507 | 2,656 | 41100 | …taccttccag | gtggcatatg… | …gatctttgac | gtgagtgggg… | 0.251 |
| 15 | 171 | 2,908 | 3,078 | 41100 | …ccccctgcag | ttcaacagct… | …cattgagaag | gtgctgcctc… | 0.606 |
| 16 | 310 | 3,685 | 3,994 | 41100 | …ctaccctcag | cccatgggca… | …gctgatgcac | gtaagtagag… | 0.272 |
| 17 | 68 | 4,267 | 4,334 | 41100 | …cctccctcag | ctgtcgagaa… | …tctgcacagg | gtaagcatgt… | 0.365 |
| 18 | 88 | 4,700 | 4,787 | 41100 | …tggaccacag | gaaaatctga… | …aagtctccag | gtaaggccca… | 0.704 |
| 19 | 118 | 5,492 | 5,609 | 41100 | …actgctgcag | gggtgataga… | …tcaggcagag | gtgggtacag… | 0.147 |
| 20 | 124 | 5,757 | 5.880 | 41100 | …tcctctccag | gtaccgcatc… | …ccacaccaag | gtgagggaag… | 0.261 |
| 21 | 137 | 6,142 | 6,278 | 41100 | …ttggctatag | gtgttcttca… | …tggaacgcag | gtgagagctt… | 0.240 |
| 22 | 256 | 6,519 | 6,774 | 41100 | …gcccctgcag | agactccttg… | …agtgcaagcg | gtgaggctcc… | 0.609 |
| 23 | 243 | 7,384 | 7,626 | 41100 | …ttgtcctcag | gaacaagaca… | …agagaacaag | gtgagggcaa… | 0.149 |
| 24 | 177^2^ | 7,776 | 7,953 | 41100 | …tgttcggcag | gtgaagaacc… | …ggtggatgat | gtaagtagat… | 0.783 |
| 25 | 146 | 8,737 | 8,882 | 41100 | …cccccaccag | ctggagggat… | …ggcttaaaaa | gtagagtgtt… | 1.098 |
| 26 | 91 | 9,981 | 10,071 | 41100 | …ttctccacag | gaaggacttt… | …agagcttcag | gtgaggtgtg… | 0.865 |
| 27 | 390 | 10,936 | 11,325 | 41100 | …cccggcccag | gctcggatcg… | …caaggccaag | gtaggctgcc… | 0.225 |
| 28 | 127 | 11,551 | 11,677 | 41100 | …cctccctcag | gctaacctgg… | …actgagaatg | gtgagcccca… | 0.175 |
| 29 | 119 | 11,853 | 11,971 | 41100 | …gcccctccag | gtgagctgtc… | …ggaggttaag | gtgaggccta… | 0.744 |
| 30 | 197 | 12,716 | 12,912 | 41100 | …ccctccacag | gcgaagaatg… | …aggaggccaa | gtgagtccca… | 0.549 |
| 31 | 184 | 13,462 | 13,645 | 41100 | …tcctgggcag | gaagaagctg… | …ctttgacaag | gtgggcctgg… | 0.189 |
| 32 | 166 | 13,835 | 14,000 | 41100 | …gtccccacag | atcctggccg… | …aaccttcagg | gtgcgctggg… | 0.146 |
| 33 | 125 | 14,147 | 14,271 | 41100 | …cctcccccag | aggagatctc… | …ggaggctgag | gtgtgtgtgc… | 0.577 |
| 34 | 309 | 14,849 | 15,157 | 41100 | …ctcctcccag | gcctctctgg… | …cttgctgaag | gtacatggag… | 0.248 |
| 35 | 204 | 13,406 | 15,609 | 41100 | …tgatctacag | gacacccaga… | …gcactcccag | gtgagtgact… | 0.129 |
| 36 | 126 | 15,729 | 15,854 | 41100 | …tgatcctcag | aataccagcc… | …catcacagat | gtgagtgact… | 0.196 |
| 37 | 276 | 16,051 | 16,326 | 41100 | …ccgtccccag | gctgccatga… | …gacttaccag | gtgcgggcat… | 0.875 |
| 38 | 96 | 17,202 | 17,297 | 41100 | …ccacccccag | acggaagagg… | …ggaggaggca | gtgagtggcc… | 0.106 |
| 39 | 135 | 17,404 | 17,538 | 41100 | …acccccacag | gaagagcagg… | …tggcgccaag | gtgggtccct… | 0.589 |
| 40 | 18 ORF | 18,128 | (18,145) | 41100 | …ctttcaaaag | ggcttgaatg… |  |  |  |

^1^ the estimated distance between [Genbank: AAEX01041099] and [Genbank: AAEX01041100] was 426 bp; ^2^according to build 1.1 of the canine genome, exon 24 counted 178 bp. However, bp G at position 7.902 of [Genbank: AAEX01041100] (exon 24) was not present in any of 11 *Canis familiaris* trace sequences covering this position, it disturbed the reading frame and did not seem to be correct. Therefore, the assumed size of exon 24 was 177 bp.

Additional file 1H

Intron-exon structure of canine *PLN*

| Exon | Exon size | Location | | Gen.contig | Intron | 5'end exon | 3'end exon | Intron | Intron |
| --- | --- | --- | --- | --- | --- | --- | --- | --- | --- |
| no. | (bp) | from bp | to bp | AAEX010..... |  |  |  |  | size(Kb) |
| 2 | 159 ORF | (52,619) | (52,461) | 14037 |  |  |  |  |  |

Additional file 1J

Intron-exon structure of canine *SGCD*

| Exon | Exon size | Location | | Gen.contig | Intron | 5'end exon | 3'end exon | Intron | Intron |
| --- | --- | --- | --- | --- | --- | --- | --- | --- | --- |
| no. | (bp) | from bp | to bp | AAEX010..... |  |  |  |  | size(Kb) |
| 3 | 189 ORF | (21,671) | 21,483 | 16852 | …tttatttcag | atgcctcagg… | …cttcacaatt | gtaagtaaaa… | 149.375^1^ |
| 4 | 102 | 7,767 | 7,666 | 16851 | …tctatttcag | gatggaatgg… | …gtcccgacca | gtaagtactg… | 79.568^2^ |
| 5 | 88 | 157,785 | 157,698 | 16848 | …gtcattacag | ggtaatgccc… | …ctcataacag | gtaagaaaac… | 5.564 |
| 6 | 120 | 152,133 | 152,014 | 16848 | …ttccttctag | gtccaaaagc… | …agagttttag | gtaaggaaac… | 35.618 |
| 7 | 73 | 116,395 | 116,323 | 16848 | …ttgtttacag | gagcggaggg… | …aggaactaag | gtaaacttct… | 84.423 |
| 8 | 124 | 31,899 | 31,776 | 16848 | …tgtatttcag | gttggagtcc… | …agatggagag | gtaagggtac… | 1.397 |
| 9 | 174 ORF | 30,378 | (30,205) | 16848 | …gtttctttag | attaagttag… |  |  |  |

^1^the estimated distance between [Genbank: AAEX01016852] and [Genbank: AAEX01016851] was 321 bp; ^2^the estimated distance between [Genbank: AAEX01016848] and [Genbank: AAEX01016849] was 781 bp, between [Genbank: AAEX0106849] and [Genbank: AAEX0106850] was 200 bp, between [Genbank: AAEX0106850] and [Genbank: AAEX0106851] was 1 bp, and between [Genbank: AAEX0106851] and [Genbank: AAEX0106852] was 321 bp.

Additional file 1K

Intron-exon structure of canine *TCAP*

| Exon | Exon size | Location | | Gen.contig | Intron | 5'end exon | 3'end exon | Intron | Intron |
| --- | --- | --- | --- | --- | --- | --- | --- | --- | --- |
| no. | (bp) | from bp | to bp | AAEX010..... |  |  |  |  | size(Kb) |
| 1 | 110 ORF | (29,389) | 29,498 | 22011 |  |  | …ctgaggaggg | gtgagtgcca… | 0.223 |
| 2 | 394 ORF | 29,722 | (30,115) | 22011 | …tcctccccag | ttgctctctg… |  |  |  |

Additional file 1L

Intron-exon structure of canine *TNN-I3*

| Exon | Exon size | Location | | Gen.contig | Intron | 5'end exon | 3'end exon | Intron | Intron |
| --- | --- | --- | --- | --- | --- | --- | --- | --- | --- |
| no. | (bp) | from bp | to bp | AAEX010..... |  |  |  |  | size(Kb) |
| 1 | 11 ORF | (5,916) | 5,906 | 53923 |  |  | …tggcggatga | gtgagtgcat… | 0.235 |
| 2 | 13 | 5,670 | 5,658 | 53923 | …cttccttcag | gagcggcgatgcg |  | gtgagaggag… | 0.138 |
| 3 | 87 | 5,519 | 5,433 | 53923 | …ccaaccccag | gcggggtgcc… | …gcacgccaag | gtgagcgagg… | 0.364 |
| 4 | 42 | 5,068 | 5,027 | 53923 | …accccggcag | aaaaagtcca… | …gcagctgaag | gtgctggtgg… | 0.203 |
| 5 | 132 | 4,724 | 4,593 | 53923 | …gcctctgcag | accctgatgc… | …ggagctgcag | gtaccgttct… | 0.375 |
| 6 | 90 | 4,217 | 4,128 | 53923 | …tcttgaccag | gacttgtgcc… | …catcacagag | gtggggggcc… | 0.497 |
| 7 | 177 | 3,630 | 3,454 | 53923 | …ctgcccccag | atagcagatc… | …cacagagaag | gtgagtgtgg… | 0.425 |
| 8 | 84 ORF | 3,028 | (2,945) | 53923 | …attcctccag | gaaaaccggg… |  |  |  |

Additional file 1M

Intron-exon structure of canine *TNN-T2*

| Exon | Exon size | Location | | Gen.contig | Intron | 5'end exon | 3'end exon | Intron | Intron |
| --- | --- | --- | --- | --- | --- | --- | --- | --- | --- |
| no. | (bp) | from bp | to bp | AAEX010..... |  |  |  |  | size(Kb) |
| 1 |  | no hits^1^ |  |  |  |  |  |  |  |
| 2 | 11 | 794 | 804 | 13360 | …ttgcacgcag | ggagcaggaa | g | gtaagcacac… | 0.101 |
| 3 | 15 | 906 | 920 | 13360 | …tcctgagcag | aagaagctgt | ggaag | gtaggagccc… | 2.377 |
| 4 | 30 | 3,298 | 3,327 | 13360 | …cccccagaag | aagaggagga… | …cacgacgacg | gtagtacagc… | 1.881 |
| 5 | 66 | 5,209 | 5,274 | 13360 | …cgccctccag | agcaggaaga… | …aacgcggaag | gtaaagcctg… | 0.311 |
| 6 | 36^2^ | 5,586 | 5,622 | 13360 | …ttctgtgcag | gagatgccca… | …gaggccgaagg | gtgagggctt… | 0.872 |
| 7 | 34 | 6,495 | 6,528 | 13360 | …tcctctccag | atggcccggt… | …caaagcccag | gtgagtgggc… | 1.023 |
| 8 | 61 | 7,552 | 7,612 | 13360 | …tgtcccacag | gccattcatg… | …agactttgac | gtgagcgtgc… | 0.259 |
| 9 | 117 | 7,872 | 7,988 | 13360 | …gcgtccgcag | gacatccacc… | …agacaggatt | gtgagtgtcc… | 0.741 |
| 10 | 78 | 8,730 | 8,807 | 13360 | …tcccccgtag | gagaagcggc… | …tcgcctggct | gtgagtgacc… | 1.614 |
| 11 | 111 | 10,422 | 10,532 | 13360 | …tgctaaccag | gaggagagag… | …catccagaag | gtgggccggg… | 0.730 |
| 12 | 6 | 11,263 | 11,268 | 13360 | …ggctctgcag | gcccag |  | gtgggtacag… | 0.334 |
| 13 | 108^3^ | 11,603 | 11,701 | 13360 | …ccctctccag | accgagcgga… | …accagctg.. |  | 0.867^4^ |
| 14 | 91 | 406 | 496 | 13359 | …ggccgcgcag | ggagaaggcc… | …caaatacgaa | gtgagctgct… |  |
| 15 |  | no hits^1^ |  |  |  |  |  |  |  |
| 16 |  | no hits^1^ |  |  |  |  |  |  |  |

^1^exon could not be identified in build 1.1 of the canine genome. Considering the sizes of the respective exon and adjacent intron of human *TNNT2*, the exon may be located in a region still unsequenced; ^2^ according to build 1.1 of the canine genome, exon 5 counted 37 bp. However, bp G at position 5,622 of [Genbank: AAEX01013360], the last bp of exon 5 (underlined), was not present in the 2 *Canis familiaris* trace sequences covering this position, it disturbed the reading frame and did not seem to be correct. Therefore, the assumed size of exon 5 was 36 bp ORF; ^3^Genomic contig [Genbank: AAEX01013360] (11,701 bp) stopped 2 bp before the end of exon 12 (in comparison to the human exon 12), therefore, only 108 bp sequence of the exons’ expected 110 bp were available; ^4^the estimated distance between [Genbank: AAEX01013360] and [Genbank: AAEX01013359] was 779 bp.

Additional file 1N

Intron-exon structure of canine *TPM1*

| Exon | Exon size | Location | | Gen.contig | Intron | 5'end exon | 3'end exon | Intron | Intron |
| --- | --- | --- | --- | --- | --- | --- | --- | --- | --- |
| no. | (bp) | from bp | to bp | AAEX010..... |  |  |  |  | size(Kb) |
| 1 | 114 ORF | (60,384) | 60,497 | 08742 |  |  | …gagcaagcag | gtctgcgcct… | 1.121 |
| 2 | 126 | 61,620 | 61,745 | 08742 | …caactcccag | ctggaagatg… | …ggccactgac | gtaagtgcac… | 12.571 |
| 3 | 134 | 74,317 | 74,450 | 08742 | …ctctgcctag | gctgaagccg… | …agagcgagag | gtgagggtgc… | 2.482 |
| 4 | 118 | 76,933 | 77,050 | 08742 | …tacttttcag | aggcatgaaa… | …gtatgaagag | gtcagatctt… | 1.295 |
| 5 | 71 | 78,346 | 78,416 | 08742 | …tgttctacag | gtggcccgta… | …tctcagaaag | gtaagtaggc… | 0.256 |
| 6 | 76 | 78,673 | 78,748 | 08742 | …ccactaacag | ccaagttcga… | …agaggataag | gtactgatgg… | 0.967 |
| 7 | 63 | 79,716 | 79,778 | 08742 | …ctaattacag | tactcgcaga… | …gctgaaggag | gtaatatgag… | 0.276 |
| 8 | 70 | 80,055 | 80,124 | 08742 | …atcattgcag | gccgagactc… | …gacttagaag | gtaagatctt… | 1.429 |
| 9 | 83 ORF | 81,554 | (81,636) | 08742 | …ctggtcatag | acgagctgta… |  |  |  |

Additional file 1P

Intron-exon structure of canine *VCL*

| Exon | Exon size | Location | | Gen.contig | Intron | 5'end exon | 3'end exon | Intron | Intron |
| --- | --- | --- | --- | --- | --- | --- | --- | --- | --- |
| no. | (bp) | from bp | to bp | AAEX010..... |  |  |  |  | size(Kb) |
| 1 | 168 ORF | (63,657) | 63,824 | 16404 |  |  | …ccttgtccgg | gtgagcgcgc… | 41.010 |
| 2 | 71 | 104,835 | 104,905 | 16404 | …tctcctatag | gttggaaaag… | …catttattaa | gtgagtgatt… | 27.568 |
| 3 | 151 | 132,474 | 132,624 | 16404 | …cttcccacag | ggttgagaat… | …tgaggctgag | gtaggcaatc… | 0.208 |
| 4 | 109 | 132,833 | 132,941 | 16404 | …atgtttacag | gttcgtaaaa | …cttggaccag | gttagttgcg… | 1.902 |
| 5 | 123 | 134,844 | 134,966 | 16404 | …tatgcctcag | gaatgactaa | …ctcatttcag | gtattttctg… | 1.755 |
| 6 | 161 | 136,722 | 136,882 | 16404 | …ttttttatag | ctatgaagat… | …ggccagcaag | gttagtgtta… | 8.645 |
| 7 | 91 | 145,528 | 145,618 | 16404 | …tcttcaaaag | gacactgaag… | …gcctctccag | gtaatctagt… | 0.810 |
| 8 | 148 | 146,429 | 146,576 | 16404 | …tttattttag | gggatgctgg… | …tccgagccag | gtaaaattcc… | 3.244 |
| 9 | 154 | 149,821 | 149,974 | 16404 | …ttttgaacag | aggacaagga… | …tgctgctcag | gtagtaatga… | 0.688 |
| 10 | 176 | 150,663 | 150,838 | 16404 | …gtgtgtgcag | aattggcttg… | …tgcggagaca | gtatgtattt… | 3.266 |
| 11 | 191 | 154,105 | 154,295 | 16404 | …ttctcttcag | gggcaaagga… | …cggggtgttg | gtaaggccat… | 1.802 |
| 12 | 200 | 156,098 | 156,297 | 16404 | …ctggatccag | gtcaggctgc… | …ctccttaaag | gtaaaacttg… | 1.155 |
| 13 | 129 | 157,453 | 157,581 | 16404 | …tgtttttaag | gatctgaaag… | …tagggaggaa | gtgggtatct… | 4.961 |
| 14 | 150 | 162,543 | 162,692 | 16404 | …tttcctcaag | gtatttgatg… | …cacaccccag | gttgaatttt… | 2.361 |
| 15 | 109 | 165,054 | 165,162 | 16404 | …tcatccacag | gtggtctcag… | …aaaatgacag | gtagggttgt… | 1.118 |
| 16 | 303 | 166,281 | 166,583 | 16404 | …ctcctgacag | ggctggtgga… | …tccgaccctg | gtaagcaatg… | 1.255 |
| 17 | 125 | 167,839 | 167,963 | 16404 | …ttatgtttag | gcctgcaaaa… | …acagctccgt | gtaagtaaat… | 1.285 |
| 18 | 186 | 169,249 | 169,434 | 16404 | …tcttccttag | ctggcagatg… | …gtccagcaag | gtaagtaagg… | 2.872 |
| 19 | 204 | 172,307 | 172,510 | 16404 | …atcgaccaag | ccgggtaacc… | …gtctagtaag | gtactgatta… | 1.985 |
| 20 | 204 | 174,496 | 174,699 | 16404 | …ttctctctag | ggcaatgaca… | …cctcttacag | gtactcaaga… | 0.392 |
| 21 | 105 | 175,092 | 175,196 | 16404 | …tcctttttag | gtatgcgagc… | …gtctgagcag | gtatgtgtcc… | 2.165 |
| 22 | 147 ORF | 177,362 | (177,508) | 16404 | …ttgcttgcag | gccacagaga… |  |  |  |
